# Genomic Surveillance Reveals Vaccine-Associated Shifts in Pediatric Invasive *Streptococcus pneumoniae* in Tunisia

**DOI:** 10.3390/vaccines14010027

**Published:** 2025-12-25

**Authors:** Samar Mhimdi, Khaoula Meftah, Ala-Eddine Deghmane, Yasmine Chelbi, Aida Bouafsoun, Muhamed-Kheir Taha, Hanen Smaoui

**Affiliations:** 1 Laboratory LR18ES39, Faculty of Medicine of Tunis, University of Tunis El Manar, Tunis 1007, Tunisia; meftahkhaoula@gmail.com (K.M.); hanen.smaoui@gmail.com (H.S.); 2 Microbiology Laboratory, Children’s Hospital Bechir Hamza of Tunis, Tunis 1006, Tunisia; chelbiyesmine@gmail.com (Y.C.); aida.bouaf@gmail.com (A.B.); 3 Invasive Bacterial Infections Unit, National Reference Centre for Meningococci and Haemophilus Influenza, Institut Pasteur, Cedex 15, 75724 Paris, France; ala-eddine.deghmane@pasteur.fr (A.-E.D.); muhamed-kheir.taha@pasteur.fr (M.-K.T.)

**Keywords:** *Streptococcus pneumoniae*, pediatric IPD, serotyping, drug resistance, genetic population structure, vaccination impact, *k*-mers

## Abstract

**Background/Objectives**: *Streptococcus pneumoniae* (*S. pneumoniae*) remains a leading cause of invasive bacterial disease in children worldwide. In Tunisia, the 10-valent pneumococcal conjugate vaccine (PCV10) was introduced into the national immunization program in 2019 for children under two years of age. This study aimed to assess molecular epidemiology, antimicrobial resistance, and vaccine impact on pediatric invasive pneumococcal disease (IPD) before and after PCV10 introduction. **Methods**: A retrospective study was conducted at Bechir Hamza Children’s Hospital (Tunis, Tunisia) between 2016 and 2022. IPD isolates were characterized by multiplex PCR, antimicrobial susceptibility testing, and whole-genome sequencing. Serotyping was performed using three approaches: multiplex PCR, SeroBA, and a novel *cpsB* gene-based algorithm. Genomic diversity and population structure were analyzed through molecular typing approaches. Incidence trends were calculated using national population data. **Results**: Among 150 confirmed IPD isolates, vaccine-type (VT-PCV10) strains decreased significantly from 69.8% before to 47.2% after vaccine introduction (*p* = 0.013), with serotype 14 showing the largest decline. Genomic analysis identified 43 sequence types and 27 global pneumococcal sequence clusters, reflecting high genetic heterogeneity. The *cpsB* approach demonstrated strong concordance with PCR (κ = 0.67) and SeroBA (κ = 0.85). The mean annual incidence of VT disease in children aged 0–4 years declined from 1.28 to 0.86 cases per 100,000 population, while non-vaccine serotypes showed a modest increase. **Conclusions**: PCV10 introduction was associated with a marked reduction in vaccine-type IPD among young children, supporting its public health benefit. Ongoing genomic surveillance remains essential to monitor serotype replacement and antimicrobial resistance in Tunisia.

## 1. Introduction

*Streptococcus pneumoniae* is a major human pathogen and a leading cause of childhood morbidity and mortality worldwide, responsible for a wide spectrum of severe infections including meningitis, pneumonia, and bacteremia [1,2]. The World Health Organization estimates that pneumococcal infections cause approximately one million deaths annually among children under five years, mostly in low- and middle-income countries [3,4].

The polysaccharide capsule is the principal virulence determinant of *S. pneumoniae* and the basis for its classification into more than 100 serotypes [5]. Despite this diversity, a limited subset of pneumococcal serotypes, including serotypes such as 1, 3, 5, 6A, 6B, 19F, 14, 19A, and 23F, accounts for most invasive infections and is often associated with high levels of antimicrobial resistance [6]. The extensive serotype variation facilitates persistence in human populations and immune evasion, posing major challenges for vaccine design and disease control [7].

Antimicrobial resistance in *S. pneumoniae* has risen globally over recent decades, complicating treatment and control [8]. The frequent and sometimes inappropriate use of antibiotics has driven the selection and spread of multidrug-resistant strains. Tunisia ranks among the world’s highest antibiotic consumers [9,10]. Consequently, the World Health Organization lists *S. pneumoniae* among priority pathogens for which new strategies are urgently needed [11].

To address this burden, pneumococcal conjugate vaccines were initially introduced in Tunisia’s private sector, starting with PCV7 in 2008, followed by PCV13 and PCV10. In April 2019, PCV10 (Synflorix^®^, GSK), covering 10 serotypes (vaccine-type) [1, 4, 5, 6B, 7F, 9V, 14, 18C, 19F, and 23F], was added to the national immunization program for children under two years [12,13]. The other serotypes are classified as non-vaccine-type. In Tunisia, PCV10 vaccination coverage among children was already high at the early stages of vaccine implementation, with the completion rate of the 3-dose schedule (PCV3) reaching 87% in 2020 and increasing to 98% by 2022. Data prior to 2020 were not officially recorded in the national data of the National Immunization Program [14]. While PCV implementation worldwide has substantially reduced vaccine-type invasive pneumococcal disease (VT-IPD), this success has been partly offset by the emergence of non-vaccine serotypes, many linked to increasing multidrug resistance [15,16].

Ongoing genomic surveillance is critical to detect such serotype replacement and guide vaccine policy. Whole-genome sequencing (WGS) has revolutionized pneumococcal epidemiology by enabling high-resolution analysis of genetic diversity, clonal lineages, and serotype prediction [17,18]. WGS provides a powerful framework to track national and international clones, study transmission dynamics, and monitor vaccine-related shifts in circulating lineages.

This study aimed to investigate the phenotypic and genomic characteristics of *S. pneumoniae* isolates causing pediatric invasive disease in Tunisia before and after PCV10 introduction. We assessed changes in serotype distribution, antimicrobial resistance, and population structure, providing a genomic overview of pneumococcal evolution in the post-vaccine era.

## 2. Materials and Methods

### 2.1. Clinical Isolates and Study Cohort

A retrospective study was conducted at the Microbiology Laboratory of Bechir Hamza Children’s Hospital (BHCH) in Tunis, Tunisia, covering a seven-year period from January 2016 to December 2022. The study included cases of invasive pneumococcal disease (IPD), defined by the isolation of *Streptococcus pneumoniae* from sterile clinical sites—blood, cerebrospinal fluid (CSF), pleural fluid (PF), or joint fluid (JF)—confirmed by PCR.

Only non-redundant isolates were included, excluding duplicates from the same patient and infection episode. Children under 15 years hospitalized in BHCH were eligible. Demographic and clinical data were collected from patient medical records using a standardized form. Population data were obtained from the National Institute of Statistics “https://www.ins.tn/ (accessed on 21 October 2025)”. Antimicrobial resistance data were retrieved from the SIRscan system archives (i2a-diagnostics^®^, Montpellier, France). All confirmed *S. pneumoniae* isolates were stored at −80 °C for further analysis. The study period was divided into two vaccine eras: P1, the pre-vaccine period (2016–April 2019), and P2, the post-vaccine period (2020–2022).

### 2.2. Bacterial Culture and Identification

Isolates were cultured on 5% sheep blood agar (bioMérieux^®^, Marcy-l’Étoile, France) and incubated overnight at 37 °C in 5% CO_2_. Preliminary identification was based on colony morphology, alpha-hemolysis, Gram staining, and optochin susceptibility.

Genomic DNA for PCR was extracted by the heat lysis method. Bacteria were harvested from plates and resuspended in bi-distilled water. After a freeze–thaw cycle, the bacterial suspension was boiled, and the supernatant was recovered after centrifugation and quantified using a Nano spectrophotometer (Jenway™, Genova Nano, Dunmow, UK). Molecular confirmation was performed by PCR targeting the *cpsA* gene, encoding the capsular polysaccharide synthesis protein [19]. To ensure accurate species identification, isolates negative for *cpsA* were further screened for two *S. pneumoniae*-specific genes, *lytA* (gene encoding the major autolysin A) and *piaA* (gene encoding a component of the iron-uptake ABC transporter system) [20].

### 2.3. Antimicrobial Susceptibility Testing

Antimicrobial susceptibility testing followed the recommendations of the Antibiogram Committee of the French Society for Microbiology (CA-SFM) and the European Committee on Antimicrobial Susceptibility Testing (EUCAST). Screening for penicillin non-susceptible pneumococci (PNSP) used a 1 µg oxacillin (OXA) disk.

The following antibiotics were tested: oxacillin (1 µg), tetracycline (30 µg), chloramphenicol (30 µg), trimethoprim–sulfamethoxazole (25 µg), erythromycin (15 µg), lincomycin (15 µg), pristinamycin (15 µg), rifampin (30 µg), and vancomycin (30 µg). Minimum inhibitory concentrations (MICs) for penicillin, amoxicillin, and cefotaxime were determined using E-test strips (bioMérieux^®^, France). *S. pneumoniae* ATCC 49619 served as the quality control strain. Isolates resistant to ≥3 antimicrobial classes were classified as multidrug resistant (MDR) [21].

### 2.4. Whole-Genome Sequencing (WGS)

Whole-genome sequencing was performed at the Institut Pasteur (Paris, France) in the Invasive Bacterial Infections Unit. DNA was extracted using the NucleoMag^®^ DNA Bacteria Kit (Macherey-Nagel, Düren, Germany). Libraries were prepared with the Nextera XT DNA Library Preparation Kit (Illumina, San Diego, CA, USA) and sequenced on the Illumina NextSeq^®^ 500 platform.

Genome assemblies were generated using SPAdes v3.15.5 (CAB, Saint Petersburg State University, Saint Petersburg, Russia). Species confirmation and typing were performed on PubMLST using ribosomal MLST (rMLST) for species identification and allelic MLST profiles for sequence typing. Phylogenetic relationships were inferred using SplitsTree v4.14.6 (University of Tübingen, Germany) and multiple sequence alignments generated with CLUSTALW v2.1. Population structure analysis was performed using Pathogenwatch (PW) online tool v13.0.0 “https://pathogen.watch/ (accessed on 16 September 2025)”.

High-quality sequence assemblies were selected for genomic analysis and genomic-based serotyping. The following criteria from the tools on PubMLST.org were used to check the completeness of the assembly: rMLST score > 90%, Genome length > 1.9 Mb, GC content 39% to 41%, N50 > 30 Kb, and number of contigs < 200. Core genome MLST (cgMLST) analysis was conducted using GrapeTree [22] (available on https://pubmlst.org/), and Life Identification Number (LIN codes) of the isolates were obtained from pubmlst.org.

### 2.5. Pneumococcal Isolate Serotyping

Capsular typing of *S. pneumoniae* isolates was carried out using a series of conventional multiplex PCR assays targeting the most prevalent serotypes circulating in Tunisia. The panel included the following serotypes and serogroups: 1, 3, 4, 5, 6A, 6B, 7F, 8, 9V, 14, 18C, 19F, 19A, 23F, 6C, 6D, 12F, 15A, 15B/C, 9N/L, 10A, 11A/D, 16F, 17F, 20, 22F/A, 23A, 23B, 24, 33F/37, 35F, and 35B [23]. Each multiplex PCR reaction was designed to yield two bands in the electrophoresis profile: a species-specific positive control band corresponding to the *cpsA* gene (capsular polysaccharide A), and a serotype-specific amplicon. The typing protocol followed guidelines established by the Centers for Disease Control and Prevention (CDC) and the World Health Organization [23].

SeroBA was also used for serotyping. It is integrated into the pneumococcal genome analysis workflow on PW. It can rapidly predict pneumococcal serotypes from assembled genomes “(https://cgps.gitbook.io/pathogenwatch/technical-descriptions/typing-methods/seroba (accessed on 16 September 2025)”.

A third serotyping method was based on the analysis of the *cpsB* gene [24]. We developed a bag-of-words approach using a custom Python 3.9 script to classify DNA sequences based on *k*-mer signatures unique to known serotypes [25]. *cpsB* sequences specific for 45 serotypes were extracted as FASTA files from PUBMLST using the blast function after filtering for high-quality sequences as mentioned above, and for serotyping performed by Quellung reaction and or SeroBA (Supplementary Materials Texts S1 and S2). The selected serotypes encompass all serotypes currently circulating in Tunisia [26,27].

FASTA files were parsed using Biopython [28], and strict unique *k*-mers of several sizes were computed and compared across serotypes. To determine the optimal *k*-mer length for distinguishing between groups of sequences, we applied a theoretical and empirical optimization approach. We used the parameters δ (delta) and ε (epsilon) to control, respectively, the probability of random matches among sequences and the likelihood that a *k*-mer is observed without sequencing errors. This theoretical range was subsequently verified and adjusted empirically to the range of 20–30 nucleotides using frequency-based *k*-mer comparison and statistical significance testing (Fisher’s exact test with multiple-testing correction) [29,30].

Unknown sequences were classified by Fisher’s exact test with false discovery rate (FDR) correction using SciPy and stats models libraries [31]. Visualization was performed with matplotlib and seaborn, and a Tkinter graphical interface was implemented for interactive input and result exploration. The script is accessible at https://huggingface.co/spaces/IBIBoW/Sp_cpsB_serotyping “(accessed on 15 November 2025)”.

### 2.6. Ethical Considerations

Our study was conducted in accordance with ethical standards and approved by the local ethics committee of Bechir Hamza Children’s Hospital (BHCHT) under approval number #27/2021. The analyses were performed on bacterial strains stored at −80 °C. No identifiable personal information was collected. Patient confidentiality was strictly maintained throughout the study, and all data were collected and analyzed in an anonymized manner to ensure privacy and compliance with ethical guidelines, and individual informed consent was waived.

## 3. Results

### 3.1. Clinical Isolates of S. pneumoniae

**Figure 1:**
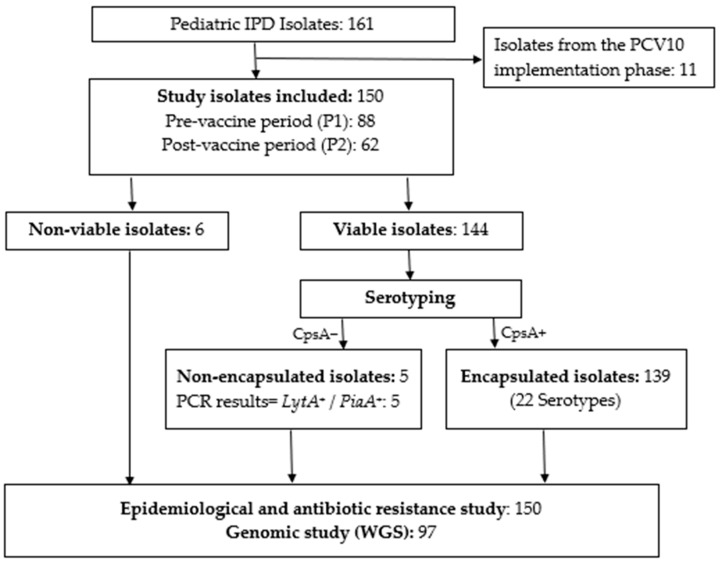
Study enrollment flowchart. IPD: invasive pneumococcal disease; WGS: whole-genome sequencing. Isolates collected during the PCV10 implementation phase were excluded from the analysis.

Between 2016 and 2022, a total of 161 cases of invasive pneumococcal disease (IPD) were recorded, including 150 cases collected during the defined pre- and post-vaccine periods: 88 in the pre-vaccine period (P1) and 62 in the post-vaccine period (P2). Eleven additional isolates were obtained during the PCV10 implementation phase. The case selection process is summarized in Figure 1.

### 3.2. Demographic and Epidemiological Features

The annual median number of IPD cases was 19. Overall, the proportion of IPD among hospitalized children (0–15 years) decreased significantly from 58.6% in P1 to 41.3% in P2 (*p* = 0.041) (Table 1).

During the COVID-19 pandemic (2020–2021), case numbers dropped sharply (15 cases total) but rebounded in 2022, when 32 cases were recorded.

A male predominance was observed (gender ratio 1.6:1), with no significant difference between periods (*p* = 0.616). Blood was the most frequent specimen source (44%), followed by cerebrospinal fluid (39%).

### 3.3. Serotype Distribution and Vaccine Coverage

**Figure 2:**
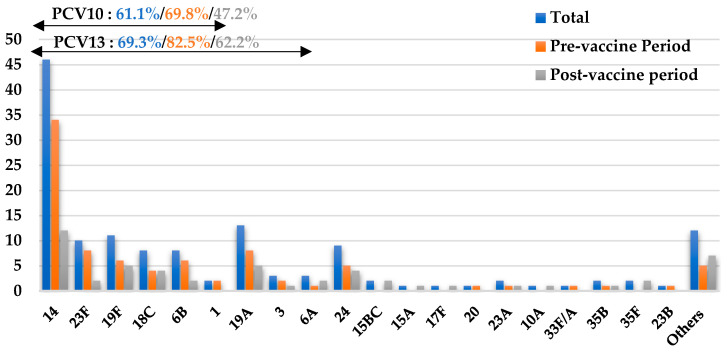
Distribution of *Streptococcus pneumoniae* serotypes in invasive pneumococcal disease. PCV10: 10-valent pneumococcal conjugate vaccine; PCV13: 13-valent pneumococcal conjugate vaccine; Others: other serotypes not included in the PCR scheme. The arrows indicate the serotypes in PCV10 and PCV13 vaccines. Theoretical vaccine coverage for PCV10 and PCV13 is indicated above the figure for the total dataset (in blue) and by period (pre- (in orange) and post-vaccine (in gray)). Coverage decreased in the post-vaccine period for both PCV10 and PCV13 vaccines.

Of 150 isolates collected across both periods, 144 were serotyped by multiplex PCR, and 139 were successfully assigned to a serotype; five were non-encapsulated (Figure 2). Across all isolates, 61.1% corresponded to PCV10 serotypes, 74.8% to PCV13, 75.5% to PCV15, and 77.7% to PCV20.

The most common PCV10 serotypes were 14 (33.1%), 19F (7.9%), and 23F (7.1%) (Figure 2). Between the two periods, vaccine-type (VT-PCV10) isolates declined significantly from 69.8% in P1 to 47.2% in P2 (*p* = 0.013), while non-vaccine types (NVT-PCV10) increased from 30.2% to 52.8% (*p* = 0.024). Only serotype 14 showed a statistically significant post-vaccine decline (*p* = 0.003), whereas other serotypes exhibited minor, non-significant changes (Figure 2).

### 3.4. Antimicrobial Resistance and Multidrug Resistance

The overall rate of penicillin non-susceptible pneumococci (PNSP) was 80.6%. MIC_90_ values for penicillin G, amoxicillin, and cefotaxime were 1.5, 3, and 0.5 mg/L, respectively. The PNSP rate increased slightly from 76.1% in P1 to 87.1% in P2 (*p* = 0.14). Among meningitis cases, penicillin resistance remained high (77.5% in P1 vs. 83.3% in P2). Amoxicillin susceptibility rose modestly (50% to 61.1%), while cefotaxime retained excellent activity (>94% susceptibility in both periods). For non-meningitis infections, high-dose penicillin susceptibility increased from 76.6% to 90.4%, with low full resistance (2.4%). Amoxicillin resistance rose slightly (10.6% to 16.7%).

During the pre-vaccine period, VT-PCV10 PNSP isolates predominated (73.1%), but their proportion declined significantly to 44.4% in P2 (*p* = 0.0025). Conversely, NVT-PCV10 PNSP increased from 25% to 48% (*p* = 0.016). Serotype 14 was the most frequent PNSP in P1 (46.3%) but decreased to 22.2% in P2 (*p* = 0.006). Resistance to other antibiotics remained stable across periods: erythromycin (77% vs. 81%), tetracycline (38% vs. 33%), trimethoprim–sulfamethoxazole (21% vs. 23%), and chloramphenicol (2.6% vs. 3%).

Overall, 58% (87/150) of isolates were multidrug resistant (MDR). MDR prevalence increased from 53.4% in P1 to 64.5% in P2 (not significant, *p* = 0.23). The most common MDR serotypes were 14 (32.1%), 19A (13.6%), 19F (11.1%), and 23F (8.6%). VT-PCV10 MDR strains decreased from 33 isolates in P1 to 16 in P2 (*p* = 0.012), while NVT-PCV10 MDR strains increased from 12 to 20 (*p* = 0.007).

### 3.5. Serotyping Comparison

After excluding non-viable or contaminated isolates, 97 genomes were retained for analysis that fulfilled the criteria for screening the quality of assembly. Serotyping was performed on 97 isolates using multiplex PCR, SeroBA, and the bag-of-words-based *cpsB* approach.

Serotype 14 was most frequent, detected in 41–45% of isolates across all methods. Other major serotypes included 23F, 19A, 24A/F, 6B, and 18C, with remaining serotypes represented by ≤2 isolates.

At the serogroup level, the methods showed strong overall concordance (Supplementary Figure S1): SeroBA vs. PCR: κ = 0.63; *cpsB* vs. PCR: κ = 0.67; SeroBA vs. *cpsB*: κ = 0.85. These findings indicate that the new *cpsB*-based method performs comparably to genomic prediction and conventional PCR serotyping.

### 3.6. Comparative Genomic Analysis and Population Structure

This analysis was also applied to the 97 genomes that were retained. MLST defined 43 sequence types (STs), with ST-2918 most prevalent (38.1%) and 33 singletons. Most STs (95%) corresponded to a single serotype, while ST-63 and ST-386 were each associated with two (15A/23F and 6A/6B) (Supplementary Table S1).

Genomic analysis identified 27 GPSCs, including 10 major lineages and 16 singletons, with one unassigned isolate. Sequences sharing the same ST were grouped within the same GPSC. GPSC6 was the predominant complex (42/97; 43%), consisting mainly of serotype 14 associated with ST-2918 (n = 37). GPSC10 was the second most frequent complex (13/97; 13%), comprising serotype 19A (ST-3772; n = 5) and serotype 24 (ST4253, n = 4; ST6227 and ST20138, n = 1 each). Other GPSCs were detected at lower frequencies: GPSC47 (n = 5), associated with serotypes 6B and 6A; GPSC9 (n = 4), linked to serotypes 23F and 15A; as well as GPSC3, GPSC12, GPSC16, GPSC132, GPSC50, and GPSC156 (2 isolates each) (Table S1).

**Figure 3:**
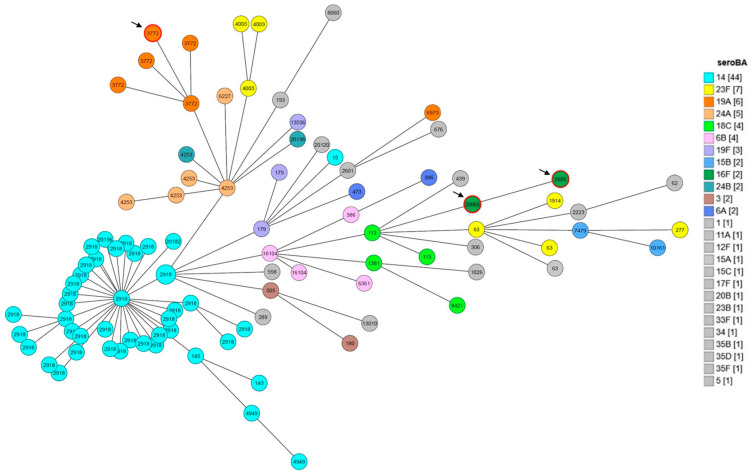
A GrapeTree based on the cgMLST loci from 97 genomes of this study. The nodes were drawn to scale according to the number of isolates (indicated by the pie chart) of each node. The nodes were colored according to serotypes as determined by SeroBA. Serotypes represented by only one isolate are all indicated in gray. The ST corresponding to each node is indicated inside the node. The branches between the nodes were drawn to the logarithmic scale, and the number of different alleles between the two connected nodes is indicated on the branch. Arrows indicate serogroup disagreement between the SeroBA and *cpsB* methods.

Although several compact clusters of isolates sharing the same serotype were apparent on the cgMLST tree, this pattern was not consistent across all lineages. In multiple parts of the tree, serotypes were intermingled, indicating that the concordance between serotype and ST is partial rather than strong. Overall, these observations suggest that serotype does not reliably capture the underlying evolutionary relationships inferred from cgMLST. The most common serotype, 14, appeared across multiple STs and LIN codes, and several other serotypes clustered closely with serotype 14 isolates (Figure 3).

### 3.7. Vaccine Impact

Following PCV10 introduction in 2019, the incidence of IPD due to vaccine serotypes (VS) declined in children aged 0–4 years, from 1.28 to 0.86 cases per 100,000 population between pre- and post-vaccine periods (2016–2018 vs. 2020–2022). In contrast, non-vaccine serotype (NVS) incidence rose slightly (0.65 to 0.72 per 100,000).

**Figure 4:**
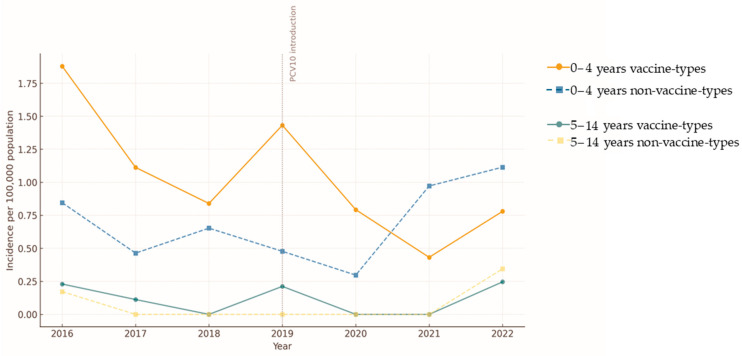
Trends in incidence of vaccine-type and non-vaccine-type pneumococcal disease before and after PCV10 introduction, Tunisia, 2016–2022. Annual incidence rates (per 100,000 population) of invasive pneumococcal disease (IPD) caused by vaccine serotypes (VS) and non-vaccine serotypes (NVS) are shown for children aged 0–4 years and 5–14 years. The vertical dashed line marks the introduction of PCV10 in 2019, targeting children under two years of age.

Among children aged 5–14 years, VS incidence remained stable (0.11 per 100,000), while NVS incidence increased modestly (0.06 to 0.09 per 100,000) (Figure 4).

These results indicate a clear decline in VS incidence among children aged 0–4 years following vaccine introduction, while a modest increase was seen in NVS-related cases (Figure 4). In the 5–14-year group, VS incidence remained stable, and NVS incidence rose slightly. These results indicate a strong direct vaccine effect with limited serotype replacement.

## 4. Discussion

This study provides the first comprehensive genomic and phenotypic characterization of *Streptococcus pneumoniae* causing pediatric invasive disease in Tunisia before and after PCV10 introduction. The findings demonstrate a clear vaccine-associated decline in vaccine-type (VT) isolates among young children, accompanied by the emergence of non-vaccine serotypes (NVTs) and sustained antimicrobial resistance.

Over the study period, a significant decrease in IPD cases was observed between P1 and P2 (*p* = 0.041), dropping from 58.6% to 41.3%. In England, a 32% decline in overall IPD incidence among children was also documented following the introduction of PCV13 in 2010 [32].

During the SARS-CoV-2 pandemic, a pronounced decline in IPD cases was observed, with only 15 cases recorded in 2020–2021. However, in 2002, case numbers rose to 32, representing a twofold increase. A similar sharp decline was reported in the Netherlands during the first and second pandemic waves, with a 50% reduction in cases between 2020 and 2022, followed by a resurgence in 2022–2023 [33]. This pattern may be linked to public health measures, such as physical distancing, masking, and improved hand hygiene, that reduced transmission of respiratory pathogens, including *S. pneumoniae* [34]. Several studies have also highlighted a post-pandemic rebound in IPD, with incidence returning to levels comparable to those seen before COVID-19 [35].

The introduction of PCV10 in Tunisia in 2019 resulted in a significant reduction in VT-PCV10 disease, particularly due to serotype 14, which declined by more than half after vaccination. Similar patterns have been reported in other regions following PCV10 and PCV13 rollout, confirming the strong direct effect of PCVs on covered serotypes [6,32,36,37,38]. The persistence of serotypes 19F and 23F, though at lower frequency, may reflect residual transmission in unvaccinated populations or incomplete indirect protection [39].

In parallel, the proportion of NVT isolates increased, driven mainly by serotypes such as 19A, 24F, 15B/C, and 33F. This expansion mirrors post-vaccine replacement dynamics observed globally, where non-vaccine serotypes rapidly occupy ecological niches vacated by vaccine types [40,41]. Although NVT disease burden remains lower than pre-vaccine VT levels, continued genomic surveillance is essential to anticipate future shifts, especially if NVTs acquire enhanced virulence or resistance traits. These trends may also indicate the future need for higher-valent conjugate vaccines to cover emerging serotypes and enhance protection against pediatric invasive pneumococcal disease.

Antimicrobial resistance in *S. pneumoniae* remains a major challenge in Tunisia, with more than 80% of isolates showing reduced susceptibility to penicillin and nearly 60% exhibiting multidrug resistance (MDR). These figures are higher than those reported in several European and North African countries, consistent with Tunisia’s high antibiotic consumption rates [9,42,43].

While the overall prevalence of PNSP and MDR isolates did not change significantly post-vaccination, their distribution shifted: VT-PCV10 resistant clones declined, whereas NVT-associated resistance increased. Similar trends have been documented after PCV introduction elsewhere [44], where replacement by resistant NVTs—such as 19A or 24F—has partially offset vaccine benefits [45,46,47]. The continued high efficacy of cefotaxime suggests that third-generation cephalosporins remain appropriate for empirical therapy of severe IPD in children [48].

Whole-genome sequencing revealed extensive genetic heterogeneity, with 43 sequence types (STs) grouped into 27 global pneumococcal sequence clusters (GPSCs). The predominance of ST-2918 and related lineages highlights the persistence of successful local clones. However, the broad genomic diversity observed, even within single serotypes, underscores the adaptive capacity of *S. pneumoniae* under vaccine and antibiotic pressure.

The two dominant lineages identified in this study (GPSC-10 and GPSC-6) are also frequently detected in countries with close travel and migration links to Tunisia, such as Morocco and France, according to the GPS database [11].

The weak correlation between serotype and genotype, as shown by cgMLST, aligns with the high recombination rates characteristic of the pneumococcal genome. This genetic plasticity facilitates capsular switching, allowing strains to evade vaccine-induced immunity while maintaining virulence and resistance determinants [41,49].

The new *cpsB* sequence-based serotyping algorithm demonstrated strong concordance with conventional PCR and SeroBA (κ = 0.85), validating its potential for genomic surveillance. Its computational efficiency and ability to analyze partial *cpsB* sequences make it a valuable tool for laboratories with limited resources or incomplete genome assemblies. Notably, the method is applicable to PCR-confirmed cases without culture. However, several disagreements were observed. These isolates require extensive genomic and phenotypic typing to better understand this disagreement.

Our study has several public health implications. The observed decline in VT-IPD supports the effectiveness of PCV10 implementation in Tunisia and highlights the importance of sustained vaccination coverage. However, the concurrent rise in NVTs and persistent antibiotic resistance emphasizes the need for continuous national surveillance and consideration of higher-valent PCVs (PCV13, PCV15, and PCV20) that cover emerging serotypes such as 19A and 24F, along with a comprehensive national strategy to combat antimicrobial resistance. Integration of genomic epidemiology into public health programs will strengthen early detection of replacement strains and resistance dissemination.

This study has some limitations. It was conducted in a single pediatric center and therefore may not fully represent the national IPD landscape. The limited number of post-vaccine isolates and the temporary decline in cases during the COVID-19 pandemic may have influenced serotype distribution trends. Nonetheless, the combination of phenotypic and genomic data provides a robust overview of pneumococcal evolution in the vaccine era.

## 5. Conclusions

The introduction of PCV10 into Tunisia’s national immunization program has significantly reduced the burden of invasive pneumococcal disease caused by vaccine serotypes in children under five years of age. This success demonstrates the vaccine’s effectiveness in a middle-income setting. However, the concurrent rise in non-vaccine serotypes and persistent antimicrobial resistance highlights the continued adaptability of *S. pneumoniae*.

Sustained genomic surveillance is therefore essential to monitor serotype replacement, detect emerging resistant lineages, and guide future vaccine strategies, including the potential adoption of higher-valent formulations such as PCV15 or PCV20.

## Figures and Tables

**Table 1:** Demographic and epidemiological characteristics of invasive pneumococcal disease (IPD) cases before and after vaccine introduction.

Features	Global Period N = 150 N (%)	Pre-Vaccine Period P1 N = 88 N (%)	Post-Vaccine Period P2 N = 62 N (%)
**Age groups** *			
[1 day-24 M[	90 (60)	58 (66)	32 (52)
[24 M-60 M[	33 (22)	15 (17)	18 (29)
≥60 M	26 (17)	14 (16)	12 (19)
**Gender**			
Male	92 (61)	52 (59)	40 (65)
female	58 (39)	36 (41)	22 (35)
**Source of specimen**			
CSF	58 (39)	40 (46)	18 (29)
Blood	66 (44)	32 (36)	34 (55)
Pleural fluid	14 (9)	7 (8)	7 (11)
Joint fluid	12 (8)	9 (10)	3 (5)

* Age missing for one patient, CSF: cerebrospinal fluid. M: month.

## Data Availability

Whole-genome sequencing data have been deposited in the PubMLST database under the *Streptococcus pneumoniae* collection. Additional data supporting this study are available from the corresponding author upon reasonable request.

## Supplementary Materials

The following supporting information can be downloaded at: https://www.mdpi.com/article/10.3390/vaccines14010027/s1, Figure S1: Agreement between pneumococcal serotyping methods at the serogroup level. Comparative analysis of *Streptococcus pneumoniae* serogroups identified by three methods: (A) SeroBA versus multiplex PCR, (B) *cpsB* sequence-based approach versus multiplex PCR, and (C) SeroBA versus *cpsB* approach. Each heatmap displays the distribution of isolates by observed (y-axis) and predicted (x-axis) serogroups, with darker shades indicating higher counts. Most isolates clustered along the diagonal, indicating high concordance among methods. Cohen’s kappa (κ) coefficients demonstrated substantial agreement between SeroBA and PCR (κ = 0.63) and between *cpsB* and PCR (κ = 0.67), and almost perfect agreement between SeroBA and the *cpsB* approach (κ = 0.85). Discrepancies were mainly observed in serogroups with few isolates or closely related capsular types (e.g., serogroups 6 and 19); Table S1: Genetic population structure of pediatric IPD isolates; Text S1: *cpsB* unknown, Text S2: *cpsB* known serotypes.
