# Supplementary material for: Genomic Surveillance Reveals Vaccine-Associated Shifts in Pediatric Invasive Streptococcus pneumoniae in Tunisia
Source: Vaccines (Basel). 2025 Dec 25;14(1):27. doi: 10.3390/vaccines14010027 (PMC12846382; doi:10.3390/vaccines14010027)
Supplement: Supplementary file 1 [file vaccines-14-00027-s001.zip › Supplementary Figure S1.pdf]

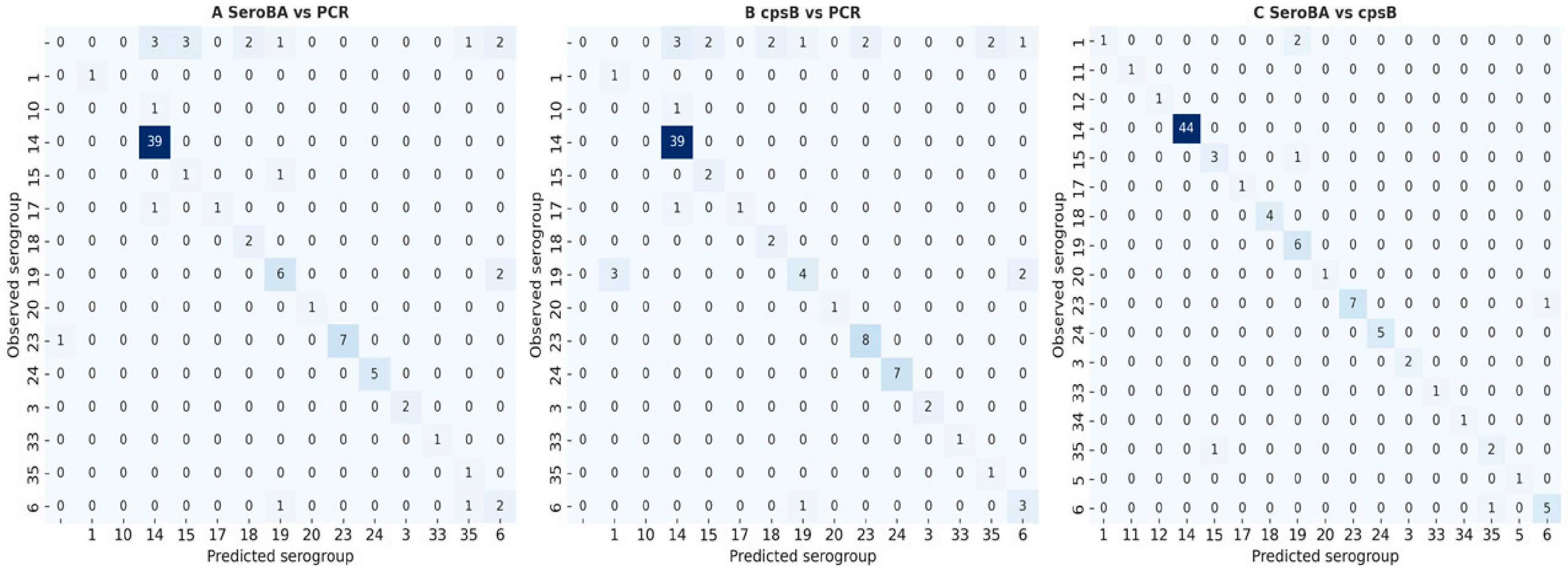

Supplementary Figure S1. Agreement between pneumococcal serotyping methods at the serogroup level.

Comparative analysis of *Streptococcus pneumoniae* serogroups identified by three methods: (**A**) SeroBA versus multiplex PCR, (**B**) *cpsB* sequence-based approach versus multiplex PCR, and (**C**) SeroBA versus *cpsB* approach. Each heatmap displays the distribution of isolates by observed (y-axis) and predicted (x-axis) serogroups, with darker shades indicating higher counts. Most isolates clustered along the diagonal, indicating high concordance among methods. Cohen's kappa (κ) coefficients demonstrated substantial agreement between SeroBA and PCR (κ = 0.63) and between *cpsB* and PCR (κ = 0.67), and almost perfect agreement between SeroBA and the *cpsB* approach (κ = 0.85). Discrepancies were mainly observed in serogroups with few isolates or closely related capsular types (e.g., serogroups 6 and 19).
