# Supplementary material for: Genomic Surveillance Reveals Vaccine-Associated Shifts in Pediatric Invasive Streptococcus pneumoniae in Tunisia
Source: Vaccines (Basel). 2025 Dec 25;14(1):27. doi: 10.3390/vaccines14010027 (PMC12846382; doi:10.3390/vaccines14010027)
Supplement: Supplementary file 1 [file vaccines-14-00027-s001.zip › Supplementary Table S1.pdf]

Supplementary Table S1. Genetic Population Structure of Pediatric IPD Isolates.

ID Identity	Year	Serotyping			ST	GPSC	LIN code
		PCR	*cpsB*	seroBA			
248944	2016	23B	23B	ND	439	7	0_57_0
248946	2016	ND	23A	16F	20066	156	0_189_1
248949	2016	14	14	14	2918	ND	0_169_0
248950	2016	19A	19A	19A	3772	10	0_15_1
248951	2016	14	14	14	2918	6	0_169_0
248952	2016	24B	24F	24A	6227	10	0_15_1
248953	2016	6B	6B	6B	386	47	0_14_0
248954	2016	ND	12F	12F	8060	55	0_10_4
248955	2016	23F	23F	23F	4003	16	0_15_0
248956	2016	18C	18C	18C	113	50	0_83_0
248957	2016	19A	1	19A	3772	10	0_15_1
248958	2016	1	1	1	306	31	0_346_0
248959	2016	19A	19A	19A	3772	10	0_15_1
249718	2016	14	14	14	2918	6	0_169_0
249719	2016	18C	18C	18C	1381	67	0_2_7
249720	2016	24B	24F	24B/24C/24F	4253	10	0_15_1
249721	2016	33A	33A	33F	2223	3	0_75_0
249722	2016	14	14	14	2918	6	0_169_0
249723	2016	14	14	14	143	6	0_169_0
249725	2016	23F	5	5	289	8	0_145_2
249726	2016	3	3	3	505	12	0_232_1
249728	2016	14	14	14	2918	6	0_169_0
249729	2016	14	14	14	2918	6	0_169_0
249730	2016	14	14	14	2918	6	0_169_0
249731	2016	14	14	14	143	6	0_169_0

249735	2017	14	14	14	2918	6	0_169_0
249736	2017	14	14	14	2918	6	0_169_0
249737	2017	14	14	14	2918	6	0_169_0
249738	2017	14	14	14	2918	6	0_169_0
249739	2017	14	11A	11A	62	3	0_75_0
249740	2017	ND	34	34	2601	213	0_46_2
249741	2017	14	14	14	2918	6	0_169_0
249742	2017	19A	19A	19A	6973	739	0_47_0
249743	2017	23F	23F	23F	277	5	0_11_3
249744	2017	14	14	14	2918	6	0_169_0
249745	2017	23F	23F	23F	4003	16	0_15_0
249747	2018	15BC	15BC	15C	193	11	0_10_0
249748	2018	24B	24F	24A	4253	10	0_15_1
249749	2018	33A	35B	35D	20120	72	0_65_0
249750	2018	19F	19F	19F	13536	10	0_15_1
249751	2018	14	14	14	2918	6	0_169_0
249752	2018	14	14	14	2918	6	0_169_0
249753	2018	14	14	14	2918	6	0_169_0
249754	2018	14	14	14	15	18	0_23_2
249755	2018	14	14	14	2918	6	0_169_0
249756	2018	24B	24F	24A	4253	10	0_15_1
249757	2018	14	14	14	4949	6	0_169_0
249758	2018	20	20	20B	1026	61	0_1_1
249941	2018	6B	6B	6B	6361	47	0_14_0
249760	2019	14	14	14	2918	6	0_169_0
249761	2019	14	14	14	2918	6	0_169_0
249762	2019	14	14	14	2918	6	0_169_0
249763	2019	14	14	14	2918	6	0_169_0

249764	2019	6B	19A	19A	3772	10	0_15_1
249942	2019	23F	23F	23F	63	9	0_74_0
249944	2019	24A	24F	24B/24C/24F	20138	10	0_15_1
249945	2019	14	14	14	2918	6	0_169_0
249946	2019	14	14	14	2918	6	0_169_0
249947	2019	18C	18C	18C	113	50	0_83_0
249948	2019	14	14	14	2918	6	0_169_0
249949	2019	23F	23F	23F	1914	9	0_74_0
249952	2019	17F	14	14	2918	6	0_169_0
249953	2019	19A	6B	6A	386	47	0_14_0
249954	2019	14	14	14	2918	6	0_169_0
249955	2019	14	14	14	2918	6	0_169_0
249956	2019	14	14	14	2918	6	0_169_0
249958	2020	ND	18C	18C	8421	67	0_2_7
249959	2020	19F	19F	19F	179	44	0_21_20
249960	2020	23F	23F	23F	63	9	0_74_0
249961	2020	14	14	14	2918	6	0_169_0
249962	2020	14	14	14	2918	6	0_169_0
249963	2020	14	14	14	2918	6	0_169_0
249964	2020	14	14	14	20156	6	0_169_0
249965	2020	14	14	14	2918	6	0_169_0
249966	2020	17F	17F	17F	13010	49	0_68_0
249967	2020	15BC	15BC	15B	7479	132	0_11_2
249969	2021	15BC	15BC	15B	10161	132	0_11_2
249971	2021	19F	19F	19F	179	44	0_21_20
249974	2021	ND	23A	16F	2685	156	0_189_1
249975	2021	6A	6B	6B	16104	47	0_14_0
249977	2021	19F	6B	6B	16104	47	0_14_0

249979	2021	6A	6A	6A	473	13	0_21_4
249980	2022	35B	35B	35B	558	59	0_99_0
255944	2022	3	3	3	180	12	0_232_1
255945	2022	10A	14	14	2918	6	0_169_0
255946	2022	14	14	14	2918	6	0_169_0
255947	2022	35F	35F	35F	676	587	0_46_3
255949	2022	23F	23F	23F	4003	16	0_15_0
255951	2022	15A	15A	15A	63	9	0_74_0
255952	2022	24A	24F	24A	4253	10	0_15_1
255953	2022	14	14	14	2918	6	0_169_0
255955	2022	19A	19A	19A	3772	10	0_15_1
255956	2022	24A	24F	24A	4253	10	0_15_1
255957	2022	14	14	14	2918	6	0_169_0
255958	2022	14	14	14	2918	6	0_169_0
255959	2022	14	14	14	20182	6	0_169_0
255960	2022	14	14	14	4949	6	0_169_0

ND: not determined
